# Draft Genome Sequence of Lactobacillus jensenii UMB0836, Isolated from the Female Bladder

**DOI:** 10.1128/MRA.00410-20

**Published:** 2020-05-21

**Authors:** Isaac Rivera, Taylor Miller-Ensminger, Adelina Voukadinova, Alan J. Wolfe, Catherine Putonti

**Affiliations:** a Department of Biology, Loyola University Chicago, Chicago, Illinois, USA; b Bioinformatics Program, Loyola University Chicago, Chicago, Illinois, USA; c Department of Microbiology and Immunology, Stritch School of Medicine, Loyola University Chicago, Maywood, Illinois, USA; d Deparment of Computer Science, Loyola University Chicago, Chicago, Illinois, USA; Portland State University

## Abstract

Lactobacillus jensenii is a frequent member of both the vaginal and urinary microbiota. Here, we present the draft genome sequence for L. jensenii UMB0836, isolated from catheterized urine obtained from a pregnant female. The genome is 1,648,234 bp long, assembled in 40 contigs.

## ANNOUNCEMENT

*Lactobacillus* species, most notably L. crispatus, L. gasseri, L. iners, and L. jensenii, inhabit both the vaginal and bladder microbiota of healthy women (1–3). Studies have shown that several of these lactobacilli play a protective role against infection, including limiting the colonization of pathogenic bacteria (4, 5). While L. gasseri and L. crispatus have been associated with urgency urinary incontinence symptoms and the lack of symptoms, respectively (6), L. jensenii has been found in the communities of individuals with and without lower urinary tract symptoms. Here, we present the draft genome of L. jensenii UMB0836, isolated from a catheterized urine sample from a pregnant woman.

L. jensenii UMB0836 was isolated as part of a prior institutional review board (IRB)-approved study (7) and cultured using the expanded quantitative urine culture (EQUC) protocol (8). Matrix-assisted laser desorption ionization–time of flight (MALDI-TOF) mass spectrometry was used to confirm the genus and species of the isolate, following a protocol previously described (8). The isolate was then stored at −80°C. The strain was streaked on a Columbia nalidixic acid (CNA) agar plate and incubated at 35°C with 5% CO_2_ for 24 h. A single colony was then grown in MRS medium supplemented with 1 ml/liter of Tween 80 under the same conditions as described above. DNA was extracted using Qiagen’s DNeasy blood and tissue kit following the manufacturer’s protocol for Gram-positive bacteria with the following exceptions: 230 μl of lysis buffer (180 μl of 20 mM Tris-Cl, 2 mM sodium EDTA, and 1.2% Triton X-100 and 50 μl of lysozyme) was used in step 2, and the incubation time in step 5 was altered to 10 min. DNA was quantified using a Qubit fluorometer. For sequencing, DNA was sent to the Microbial Genome Sequencing Center (MiGS) at the University of Pittsburgh. The Illumina Nextera kit series was used to construct the DNA library, and sequencing was performed using the NextSeq 550 platform. In total, 2,299,084 pairs of 150-bp reads were generated for this isolate. The raw reads were trimmed using Sickle v1.33 (https://github.com/najoshi/sickle) and assembled using SPAdes v3.13.0 with the “only-assembler” option for k values of 55, 77, 99, and 127 (9). The genome coverage was calculated using BBMap v38.47 (https://sourceforge.net/projects/bbmap/). PATRIC v3.6.3 (10) and the NCBI Prokaryotic Genome Annotation Pipeline (PGAP) v4.11 (11) were used to annotate the genome sequence. Unless previously noted, default parameters were used for each software tool.

The draft genome of L. jensenii UMB0836 is 1,648,234 bp long, assembled in 40 contigs, with a GC content of 34.32%. The genome coverage is 356×, with an *N*_50_ score of 63,129 bp. PGAP annotation identified 1,458 protein-coding genes, 54 tRNAs, and 7 (3 5S, 3 16S, and 1 23S) rRNA sequences. Both PGAP and PATRIC identified a single CRISPR array. This array includes 20 CRISPR spacer sequences. The draft genome sequence of L. jensenii UMB0836 provides data about the genetic content of this important member of the microbiota of the urogenital tract.

### Data availability.

This whole-genome shotgun project has been deposited in GenBank under the accession no. JAAUWN000000000. The version described in this paper is the first version, JAAUWN010000000. The raw sequencing reads have been deposited in SRA under the accession no. SRR11441036.

## References

[1] Mendes-Soares H, Suzuki H, Hickey RJ, Forney LJ 2014 Comparative functional genomics of Lactobacillus spp. reveals possible mechanisms for specialization of vaginal lactobacilli to their environment. J Bacteriol 196:1458–1470. doi:10.1128/JB.01439-13. 24488312 PMC3993339

[2] Thomas-White K, Forster SC, Kumar N, Van Kuiken M, Putonti C, Stares MD, Hilt EE, Price TK, Wolfe AJ, Lawley TD 2018 Culturing of female bladder bacteria reveals an interconnected urogenital microbiota. Nat Commun 9:1557. doi:10.1038/s41467-018-03968-5. 29674608 PMC5908796

[3] Reid G, Burton J 2002 Use of Lactobacillus to prevent infection by pathogenic bacteria. Microbes Infect 4:319–324. doi:10.1016/s1286-4579(02)01544-7. 11909742

[4] Boris S, Suárez JE, Vázquez F, Barbés C 1998 Adherence of human vaginal lactobacilli to vaginal epithelial cells and interaction with uropathogens. Infect Immun 66:1985–1989. doi:10.1128/IAI.66.5.1985-1989.1998. 9573080 PMC108154

[5] Hudson PL, Hung KJ, Bergerat A, Mitchell C 2020 Effect of vaginal Lactobacillus species on Escherichia coli growth. Female Pelvic Med Reconstr Surg 26:146–151. doi:10.1097/SPV.0000000000000827. 31990804

[6] Pearce MM, Hilt EE, Rosenfeld AB, Zilliox MJ, Thomas-White K, Fok C, Kliethermes S, Schreckenberger PC, Brubaker L, Gai X, Wolfe AJ 2014 The female urinary microbiome: a comparison of women with and without urgency urinary incontinence. mBio 5:e01283-14. doi:10.1128/mBio.01283-14. 25006228 PMC4161260

[7] Jacobs KM, Thomas-White KJ, Hilt EE, Wolfe AJ, Waters TP 2017 Microorganisms identified in the maternal bladder: discovery of the maternal bladder microbiota. AJP Rep 7:e188–e196. doi:10.1055/s-0037-1606860. 28970961 PMC5621969

[8] Hilt EE, McKinley K, Pearce MM, Rosenfeld AB, Zilliox MJ, Mueller ER, Brubaker L, Gai X, Wolfe AJ, Schreckenberger PC 2014 Urine is not sterile: use of enhanced urine culture techniques to detect resident bacterial flora in the adult female bladder. J Clin Microbiol 52:871–876. doi:10.1128/JCM.02876-13. 24371246 PMC3957746

[9] Bankevich A, Nurk S, Antipov D, Gurevich AA, Dvorkin M, Kulikov AS, Lesin VM, Nikolenko SI, Pham S, Prjibelski AD, Pyshkin AV, Sirotkin AV, Vyahhi N, Tesler G, Alekseyev MA, Pevzner PA 2012 SPAdes: a new genome assembly algorithm and its applications to single-cell sequencing. J Comput Biol 19:455–477. doi:10.1089/cmb.2012.0021. 22506599 PMC3342519

[10] Brettin T, Davis JJ, Disz T, Edwards RA, Gerdes S, Olsen GJ, Olson R, Overbeek R, Parrello B, Pusch GD, Shukla M, Thomason JA III, Stevens R, Vonstein V, Wattam AR, Xia F 2015 RASTtk: a modular and extensible implementation of the RAST algorithm for building custom annotation pipelines and annotating batches of genomes. Sci Rep 5:8365. doi:10.1038/srep08365. 25666585 PMC4322359

[11] Tatusova T, DiCuccio M, Badretdin A, Chetvernin V, Nawrocki EP, Zaslavsky L, Lomsadze A, Pruitt KD, Borodovsky M, Ostell J 2016 NCBI Prokaryotic Genome Annotation Pipeline. Nucleic Acids Res 44:6614–6624. doi:10.1093/nar/gkw569. 27342282 PMC5001611

